# Closing the Osteoporosis Care Gap: System-Level Fracture Liaison Service Implementation and a Patient Education Intervention in an Open Health System

**DOI:** 10.7759/cureus.102971

**Published:** 2026-02-04

**Authors:** Kathleena D'Anna, Clara Liu, Nasam Alfraji, Artem Minalyan, Christina Downey

**Affiliations:** 1 Rheumatology, Kaiser Permanente Fontana Medical Center, Fontana, USA; 2 Rheumatology, Loma Linda University Health, Loma Linda, USA; 3 Rheumatology, University of California Los Angeles, Los Angeles, USA

**Keywords:** fracture liaison service, open health systems, osteoporosis, osteoporosis treatment gap, quality improvement research, secondary fracture prevention, systems-based medicine

## Abstract

Background

Osteoporosis impacts millions of individuals in the United States and is linked to significant morbidity and mortality after fragility fractures. Despite the availability of effective treatments, a majority of patients remain untreated. Fracture liaison services (FLS) have been shown to improve post-fracture management, particularly in integrated healthcare systems, yet evidence from open systems remains limited. This study evaluated the impact of FLS implementation at the Loma Linda University Health (LLUH), an open healthcare system, and examined whether a patient-level educational flyer could further improve treatment initiation.

Methods

A retrospective and prospective cohort study of patients aged ≥50 years who were admitted with hip or vertebral fragility fractures between September 2015 and February 2020 was conducted. Data from September 2015 to February 2017 comprised the pre-FLS cohort. The post-FLS cohort included data from September 2017 to February 2020, excluding a six-month washout period. Data collection did not include the period following the onset of the COVID-19 pandemic in the United States. The flyer intervention was tested from 2019 to 2020, with age- and sex-matched controls receiving only standard verbal education. The primary outcome was initiation of anti-osteoporotic pharmacologic therapy within six months of hospital discharge, defined as documentation of a new prescription or medication order in the electronic health record (EHR). Secondary outcomes included completion of an outpatient follow-up visit (rheumatology or other provider) within six months of discharge and the type of anti-osteoporotic therapy initiated.

Results

A total of 404 patients were included (pre-FLS (n=216), post-FLS (n=188)). In the pre-FLS-cohort, 57% (n=124) had no documented follow-up in the EHR and 10.2% (n=22) initiated anti-osteoporotic therapy using conservative non-responder imputation. In the post-FLS cohort, 19.7% of patients initiated osteoporosis therapy, compared with 10.2% in the pre-FLS cohort. In the post-FLS cohort, patients who were seen by a rheumatologist after hospital discharge initiated therapy 57% of the time, whereas those seen by a provider outside of the LLUH system initiated therapy 40% of the time. An educational flyer Plan-Do-Study-Act initiative did not increase uptake of osteoporosis therapy (26% vs 24%, p=0.916), though outpatient rheumatology follow-up was numerically higher (59% vs 31%, p=0.235). Those seen by a rheumatologist were more likely to be treated with therapy beyond bisphosphonates.

Conclusion

Implementation of an FLS in an open health system was associated with double the odds of initiating osteoporosis treatment, although absolute rates remained low compared with those observed in closed systems. Specialist follow-up emerged as the most significant predictor of therapy initiation. The flyer intervention did not affect treatment rates, underscoring the need for integrated referral pathways and enhanced cross-network communication. Comprehensive, system-level interventions are essential to bridging the osteoporosis care gap in open health systems.

## Introduction

An estimated 10 million people in the United States have osteoporosis, an incidence higher than that of myocardial infarction, stroke, and breast cancer combined [1,2]. Often described as a silent killer, a single osteoporotic fracture not only increases the risk of subsequent lower quality of life, increased hospitalizations, dependence on mobility aids or assisted living, but also predicts higher overall mortality [3]. Current figures estimate that 20-50% of patients will require long-term care after a fracture, and the one-year mortality rate can be as high as 20-40% [4,5]. Despite the consequences of fractures and their prevalence, roughly 70% of patients will not be treated with anti-osteoporotic therapy despite the availability of safe and effective drugs [6]. In the absence of treatment, individuals with fragility fractures are at substantial risk of subsequent fractures. Twenty to 28% of patients will have an additional fracture within the five years of their first fragility fracture [7,8]. A large, US population-based cohort showed that treatment with anti-osteoporosis therapies provided a 40% age- and sex-adjusted reduction in the three-year risk of subsequent fracture [7]. 

This large care gap in the treatment of osteoporosis is multifactorial and is due to patient, clinician, and health system factors. Patient-related barriers include lack of awareness of the diagnosis, nonadherence to therapy, and an overinflated sense of potential adverse effects of treatment when compared to the risk of refracture. Clinicians may lack knowledge of therapies or their optimal timing after a fracture and may be unwilling to treat those who are older or cognitively impaired.  Potential reasons at a healthcare systems level are a lack of clear roles in caring for patients with fragility fractures and a lack of communication between the acute care and surgical teams and the outpatient care teams [6]. A healthcare system encompasses all the people, institutions, and services that contribute to care coordination, patient flows, diagnosis, disease management, and health maintenance programs. 

One way to attempt to solve the lack of role definition and communication within a health care delivery framework is to establish a Fracture Liaison Service (FLS), a strategy endorsed by the National Osteoporosis Foundation [9]. These services create an alert for a consultation with the inpatient osteoporosis care team (rheumatologists in our system) while the patient is hospitalized for a fragility fracture. Education is provided to the patient and an outpatient visit is made for the patient to follow up with a rheumatologist and initiate treatment. On average, the initiation of an FLS increases the likelihood that a patient will be treated post-fracture by up to 70%, depending on the model and healthcare setting [10]. Average rates of improvement in therapy initiation are around 35%, with larger treatment uptake in closed healthcare systems vs. open systems [10-13]. For the purposes of this study, a closed health system refers to an integrated delivery model in which inpatient, outpatient, radiology, laboratory, and pharmacy care occur within a single organization with a shared electronic health record (EHR) and insurance structure, whereas an open health system refers to a setting in which patients receive care across multiple unaffiliated providers and insurance networks with limited interoperability of medical records. 

Differences in outcomes between centers sometimes can be explained by the health insurance model in place. Systems where patients get all their care from the same healthcare entity, such as Kaiser Permanente, have shown great results from introducing an FLS, with treatment rate increases up to 65%, which they estimate has reduced the number of expected hip fractures by about 45% [14]. Kaiser Permanente is an example of a closed healthcare system. However, in open systems, where patients do not all have the same insurance plan, their outpatient physician does not have access to the medical records from the hospitalization and they may undergo barriers in their ability to follow up with an osteoporosis specialist who is in the network, the results show less robust uptake of therapy. 

Like Loma Linda University Health (LLUH), the Geisinger Health System (GHS) operates as a part of an open system with a FLS. However, Geisinger has reported substantially higher post-fracture treatment uptake following FLS implementation. The reasons for this difference remain unclear but may include variations in referral workflows, outpatient access to osteoporosis specialists, patient continuity within the system, and the degree of EHR interoperability [15]. Prior to implementation of the FLS in 2017, the treatment rate was 10%, rising to 18.6% in the year following implementation, an increase of 8.6% [16]. Understanding why FLS implementation produces variable results across open systems is critical to optimizing post-fracture care delivery. 

One idea to increase success in our system was a patient-level intervention to increase awareness of the diagnosis of osteoporosis, it’s potential consequences if left untreated and how to follow up with an osteoporosis specialist. The flyer was physically given to patients who were seen by the FLS team inpatient (a rheumatology fellow on inpatient service), was available in English and Spanish and was written at a 10th grade reading level. The flyer intervention was implemented as a predefined one-year pilot as part of a Plan-Do-Study-Act quality improvement cycle to assess feasibility and preliminary effectiveness before broader adoption.

This study was designed to address two primary research questions. First, does implementation of a FLS in an open healthcare system improve initiation of anti-osteoporotic therapy compared with pre-FLS care? Second, does the addition of a patient-level educational intervention improve outpatient follow-up or treatment initiation beyond the system-level FLS intervention alone? We hypothesized that FLS implementation would increase treatment initiation relative to pre-FLS care and that the flyer intervention would further enhance treatment initiation. 

## Materials and methods

Ethics statement

The study was approved by the LLUH Institutional Review Board. All patient data were handled in compliance with institutional privacy standards and the Health Insurance Portability and Accountability Act (HIPAA).

Study design

Methods were formulated in line with Consolidated Standards of Reporting Trials/Strengthening the Reporting of Observational Studies in Epidemiology (CONSORT/STROBE) [17] guidance for descriptive statistics. A retrospective and prospective cohort study at LLUH, a large level one academic tertiary care center serving more than one million patients annually, evaluating the effectiveness of the implementation of an FLS and subsequently employing a patient education intervention in the form of a flyer given at the bedside. Data were extracted from the Epic EHRs. To evaluate whether enhanced patient education improved care, we introduced a standardized printed educational flyer in addition to the standard verbal education provided by the rheumatology fellow performing the inpatient FLS consult. This intervention lasted one year as part of a Plan-Do-Study-Act cycle. 

This study comprised two components: 1) Systems-level FLS intervention (first objective) and 2) Patient-level flyer intervention (second objective).

Study population

Patients were identified for the first part (retrospective) of the study by reviewing hospital admissions between September 1, 2015 and February 29, 2017 using International Statistical Classification of Diseases and Related Health Problems, 10th Revision (ICD-10) codes for hip fractures (S72.0-S72.2) and vertebral fractures (M80, S22, S32). Patients were identified for the prospective aspect of the study using the same ICD-10 codes from September 1, 2017 - February 2, 2020. A six-month washout period was used to minimize contamination during initial program rollout and workflow stabilization, when referral patterns and documentation practices were evolving. The study was terminated at the onset of the COVID pandemic in the United States. If patients were admitted for multiple fragility fractures, the initial admission was analyzed. 

Those with referrals to the FLS inpatient team were included in the study. If a referral was not placed for the FLS inpatient team, the patients were not included in the study. Rheumatology fellows conducting the initial FLS intake were asked to provide the flyers to patients. Patients who received a flyer were considered the intervention group for this part of the study. An age- and sex-matched cohort of patients with FLS consults who only received verbal education were used as a control group for the prospective portion of the study. 

An exploratory subgroup analysis of the patients who were contactable after the educational flyer intervention was completed to determine if there was a difference treatment initiation or selection between groups who were seen by osteoporosis specialists (rheumatologists) or their primary care physicians (PCPs). 

Inclusion and exclusion criteria

The inclusion criteria included patients ≥50 years of age and with acute, low energy fracture (fragility fracture), defined as falling from ground level or less or vertebral fractures that were identified radiographically and judged to be fragility related. Vertebral fractures were classified as fragility-related based on radiographic reports and clinical documentation indicating low-energy mechanism or osteoporotic compression fracture, as determined by review of the medical record by the study investigators. 

The exclusion criteria included patients with high energy trauma resulting in fracture, malignancy-associated fracture, and transfers to outside hospitals.

Data collection/study measures

Data related to demographics (age, sex, race/ethnicity, language, insurance carrier), fracture location, prior fracture history, smoking status, primary care affiliations (LLUH vs. other), treatment initiated, and presence or absence of follow-up were collected.

For patients without documented outpatient follow-up in the EHR, up to three telephone contact attempts were made on separate days, with voicemail messages left when available. Patients who could not be reached after these attempts were classified as not contactable.

Outcomes

The primary outcome was initiation of anti-osteoporotic pharmacologic therapy within six months of hospital discharge, defined as documentation of a new prescription or medication order in the EHR. Secondary outcomes included completion of an outpatient follow-up visit (rheumatology or other provider) within six months of discharge and the type of anti-osteoporotic therapy initiated. The hypothesis was that outpatient follow-up care increases the likelihood of initiation of therapy as without an outpatient visit therapy can not be prescribed. A secondary end point was how many patients completed an outpatient visit but did not initiate therapy as this indicated that access to outpatient care did not guarantee uptake of anti-osteoporosis therapy. 

Further, we hypothesized that care with an osteoporosis specialist, i.e., a rheumatologist in our system, increased the likelihood of anti-osteoporosis initiation due to factors described in the introduction.

In the flyer initiation subgroup, we hypothesized that receipt of a physical flyer will increase the likelihood of treatment initiation post-discharge. 

Statistical analysis

Pre- and Post-FLS Implementation: Systems-Based Intervention

Descriptive statistics compared pre- and post-FLS cohorts. Categorical variables were analyzed using Pearson’s chi-square tests and continuous variables with independent t-tests. In exploratory analyses, logistical regression models adjusted for age, sex, race/ethnicity, fracture type and PCP affiliation to estimate the association between FLS implementation and the six-month treatment initiation. 

Patients without documented outpatient follow-up in the EHR and who were not contactable by telephone were conservatively classified as not having initiated therapy (non-responder imputation).

Patient-Level Analysis (Educational Flyer)

Intervention (flyer) and age- and sex-matched control (non-flyer) groups were compared using chi-square and t-tests. To adjust for baseline imbalances, logistical regression was performed with flyer exposure as the main independent variable and six-month treatment initiation as the dependent outcome. Age- and sex-matched controls were selected using 1:1 matching. Standardized mean differences were assessed to confirm balance between groups. Significance threshold: two-sided p<0.05 was considered statistically significant. 

## Results

Between September 2015 and February 2020, a total of 404 patients with fragility fractures were identified and included. Of these, 216 patients were in the pre-FLS group, and 188 were in the post-FLS group. The number of patients admitted to the hospital who did not receive an FLS consultation order from their primary teams was not captured by this study, but would be an area of further research as a potential area of improvement in post-fracture care. Demographics were similar in each cohort with respect to age (76.8 vs. 75.4 years, p=0.293) and sex distribution (72% vs. 70% female, p=0.651). Racial and ethnic composition differed, with more white patients in the pre-FLS group (75% vs. 55%, p<0.001). There were no other significant differences between the groups (Table 1). 

**Table 1:** Baseline characteristics of patients in the systems-level FLS intervention

		Pre-FLS (N=216)		Post-FLS (N=188)		p-value	Standardized mean differences	Test Statistic (t/χ²/F)
Average age at time of fracture		76.8		75.4		0.293	0.105	t=1.05
Female		156	72%	131	70%	0.651	0.056	χ²=0.20
Ethnicity								
Black		8	4%	13	7%	0.22	-0.144	χ²=1.52
Latin American		32	15%	43	23%	0.051	-0.207	χ²=3.82
White		163	75%	103	55%	<0.001	0.444	χ²=139
Other		13	6%	15	8%	0.564	-0.077	χ²=0.33

**Figure 1:**
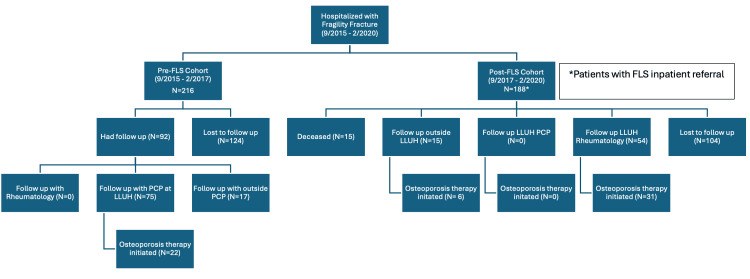
Systems-level intervention, pre and post-FLS implementation FLS, Fracture liaison service; PCP, Primary care provider; LLUH, Loma Linda University Health.

Among the 216 patients in the pre-FLS group, 43% (n=92) had documented follow-up within six months, while 57% (n=124) were lost to follow-up and were not contactable (Figure 1).

Of those who underwent follow-up, 75 patients were seen by a PCP at LLUH, 17 followed up with an outside PCP and no patients had rheumatology follow-up care. Osteoporosis therapy was initiated in 22 (24%) patients, all of whom were seen by an LLUH PCP for follow-up. When all patients without documented follow-up were conservatively classified as not having initiated therapy, the overall osteoporosis treatment initiation rate in the pre-FLS cohort was 22 of 216 patients, or 10.2%. Patients without documented follow-up in the post-FLS cohort were also handled using non-responder imputation. 

Of the 188 patients in the post-FLS cohort, 15 patients were deceased during the follow-up period. Fifteen patients completed follow-up outside of the LLUH system. Fifty-four patients completed an outpatient follow-up with an LLUH rheumatologist. No patients were seen by their LLUH PCP within the follow-up period of six months and 104 were lost to follow up. The overall osteoporosis therapy initiation rate in the post-FLS cohort was 19.7% (37 of 188 patients) when conservative non-responder imputation for patients without documented follow-up was used. The rate of treatment in patients who followed up with rheumatology in LLUH was 57% (31 of 54) and was 40% for those who followed up outside of LLUH (six of 15 patients). 

Compared with the pre-FLS cohort, the post-FLS cohort demonstrated a higher overall proportion of patients who initiated osteoporosis therapy (19.7% vs. 10.2%). Follow up with a LLUH rheumatologist was associated with a higher likelihood (17 percentage points higher) of osteoporosis therapy initiation compared to those who followed up outside the system. However, these findings are descriptive and the group sizes are small; thus, these findings should be interpreted with caution. 

There were no significant differences when comparing who followed up with an LLUH rheumatologist verses those who did not when assessing age, gender, and insurance product type. Patients were more likely to follow-up with an LLU rheumatologist if they were already a patient in the LLUH system. Patients who were seen by LLUH rheumatologists were more likely to receive biologic therapy for osteoporosis than those who received care from an outside provider. Specific treatment differences were not analyzed statistically due to the low number of patients in each group. Although treatment initiation was numerically higher among patients who followed up with LLUH rheumatology compared with those followed outside the system, these comparisons were descriptive and limited by the small sample sizes (Table 2).

**Table 2:** Post-FLS outcomes FLS, Fracture liaison service; PTH, Parathyroid hormone; LLUH, Loma Linda University Health.

		Follow-up with LLUH rheumatologist (N=54)		Follow-up outside LLUH (N=15)		Lost to follow-up (N=104)		p-value	Test statistic
Age		77		75		76		0.7	ANOVA
Female		41	76%	13	87%	75	72%	0.5	χ²	
Insurance								0.30-0.40	χ²
Medicare		29	54%	5	33%	50	48%	-	-
Medicare Advantage Plan		7	13%	5	33%	26	25%	-	-
Medicaid		9	17%	2	13%	24	23%	-	-
Commercial		9	17%	3	20%	19	18%	-	-
Established care at LLUH pre-FLS Consult?		14	26%	0	0%	10	10%	0.003	Fisher
Treatment		31	57%	6	40%	-	-	0.26	Fisher
Bisphosphonate (oral)		10	32%	6	100%	-	-	-	-
Bisphosphonate (IV)		7	23%	0	0%	-	-	-	-
Denosumab		6	19%	0	0%	-	-	-	-
PTH Analog		7	23%	0	0%	-	-	-	-
Romosozumab		1	3%	0	0%	-	-	-	-

During the intensive educational intervention, 61 patients received a physical flyer at bedside and 59 age- and sex-matched controls received verbal-only education. The cohorts were balanced in terms of demographics, however there were more hip fractures vs. vertebral fractures in the flyer group (Table 3).

**Table 3:** Baseline characteristics of the patient-level flyer intervention cohort

		Control group (N=59)		Flyer group (N=61)		p-value	Standardized mean differences	Test statistic (t/χ²/F)
Average age at time of fracture		75		75		0.99		t=1.01
Female		40	69%	42	69%	1	-0.023	χ²=0.00
Ethnicity								
Black		8	14%	6	9%	0.726	0.116	χ²=0.12
Latin American		14	24%	20	33%	0.369	-0.202	χ²=0.81
White		31	53%	30	49%	0.853	0.067	χ²=0.03
Other		6	1%	5	8%		0.068	χ²=0.07
Language								
English		53	90%	49	80%	0.229	0.269	χ²=1.46
Other		6	10%	12	20%	0.229	-0.269	χ²=1.46
Insurance								
Medicare		23	39%	22	36%	0.888	0.06	χ²=0.02
Medicaid		11	17%	10	16%	0.933	0.059	χ²=0.01
Other		25	42%	29	48%	0.7	-0.104	χ²=0.15
Smoking history		28	47%	28	46%	1	0.031	χ²=0.00
Fracture type								
Hip		43	72%	54	89%	0.052	-0.404	χ²=3.79
Vertebral		12	20%	3	4%	<0.05	0.477	χ²=6.07
Other		4	6%	4	7%	1	0.009	χ²=0.00
Previous fracture		26	44%	26	43%	1	0.029	χ²=0.00

There were no statistically significant differences in anti-osteoporotic treatment initiation between groups. Those who received the flyer were more likely to follow up with rheumatology outpatient (59% (n=26) vs. 31% (n=18); p=0.235), though this difference was not statistically significant (Table 4).

**Table 4:** Intervention group vs. control group PTH, Parathyroid hormone.

		Control group (N=59), %		Flyer group (N=61), %		p-value	Standardized mean differences	Test statistic (t/χ²/F)
Rheumatology consult at discharge by primary team		43	73%	36	59%	0.159	0.296	χ²=2.0
Rheumatology appointment completed		18	31%	26	59%	0.235	-0.254	χ²=1.4
Medication initiation		14	24%	16	26%	0.916	-0.058	χ²=0.12
Bisphosphonate		5	8%	9	15%	0.431	-0.197	χ²=0.62
Denosumab		4	6%	5	8%	0.119	0.381	χ²=2.4
PTH analog		4	6%	0	0%	1	-0.102	χ²=0.07
Romosozumab		1	1%	2	3%	1	-0.183	χ²=0.01

Despite institutional FLS protocols requiring placement of an outpatient rheumatology follow-up referral for all patients evaluated by the inpatient FLS team prior to discharge, adherence to this process occurred in only 65% of cases. 

## Discussion

Implementation of an FLS in our open health system was associated with a modest improvement in osteoporotic therapy initiation following hospitalization for a fragility fracture. Treatment rates increased from 10% to 19.7% when a conservative non-responder imputation was applied. Although this improvement did not reach the rates reported in other healthcare systems, where post-initiation rates can reach 50-65%, our findings demonstrate that system-level interventions can have a meaningful impact and partially close the post-fracture treatment gap in open systems. When compared to closed systems, open systems face challenges such as fragmented care, heterogenous insurance coverage, and a lack of communication between systems [10,14]. 

One key finding is the strong association of osteoporosis treatment initiation with a rheumatologist follow-up as an outpatient. Those seen by an LLUH rheumatologist had substantially higher rates of therapy initiation when compared to those who received care in a different setting (57% vs. 40%). While the sample sizes were small, these findings suggested that engaging with an osteoporosis specialist (in this case, a rheumatologist) may result in higher therapy adoption rates. Among patients with documented follow-up, those seen by a rheumatologist had higher rates of therapy initiation than those managed in other settings, though these findings were not statistically significant and should be interpreted cautiously. This reinforces the need for dedicated osteoporosis outpatient appointments following a fragility fracture, as demonstrated in other systems [15]. Due to system constraints, PCPs are unable to adequately address osteoporosis therapy as they are often overburdened by short appointment times and a lack of familiarity and comfort with therapy options in this high-risk group [18,19]. One limitation of the study is the lack of data for those patients served outside the EHR interoperability network. The number of patients treated could be underrepresented due to the lack of available data. Patients who followed up at outside hospitals were excluded because complete follow-up and treatment data were not reliably available; these patients may differ systematically in fracture severity or clinical complexity, though this isn't known.

The flyer directive was terminated after one year, as a Plan-Do-Study-Act cycle showed clearly that this intervention was not impactful. Medication rates were similar in both groups: 26% in the flyer group compared to 24% in the control group. This intervention relied on the rheumatology fellow remembering to hand out flyers during the FLS consult, which is less reliable than a system-level intervention. Further, education alone cannot overcome the barriers to outpatient follow-up and treatment initiation, such as insurance restrictions, limited access to in-network rheumatologists, and a lack of continuity across health systems. Interestingly, despite 120 FLS consults tracked in the flyer Plan-Do-Study-Act cycle, only 79 outpatient rheumatology referrals were placed (66%). The rheumatology referral is part of the FLS orderset in the EHR, which means the consulting services are not checking this box. One way to improve outpatient referral rates is to leave the box checked as default, rather than relying on the box to be checked during smart set placement. 

When reviewing the state of osteoporosis therapy for this project, it was noted that prior to the implementation of the FLS program most patients were treated with an oral bisphosphonate by a PCP. After implementation of the FLS, treatments began to include biologic therapies prescribed by specialists. This study was not designed to investigate the differences in specific medication initiation between osteoporosis specialists and PCPs. Further research should be done to evaluate the differences in prescribing patterns; however, the patients in this cohort are at the highest risk of refracture due to the presence of a fragility fracture [20]. Biologic therapy would be most valuable in this group as it decreases fracture incidence at much higher rates than oral alendronate. For instance, the Active‐Controlled Fracture Study in Postmenopausal Women With Osteoporosis at High Risk (ARCH) study showed that new vertebral fractures were reduced by 48% and hip fracture rates were reduced by 38% when romosozumab was compared to alendronate, while the risk of a serious cardiovascular event was only raised by 0.6% from placebo [21,22]. Survey studies have shown that PCPs are uncomfortable with comparing the likelihood of adverse effects of medications to the risk of fracture [20]. The diversity of therapies used by rheumatologists, when compared to non-osteoporosis specialists, reflect a greater familiarity with the full range of therapeutic options for osteoporosis care and supports the role of specialists in directing personalized post-fracture medical interventions. Of note, there were no patients who received anabolic therapy for post-fracture osteoporosis care while 26% of patients who were seen by a rheumatologist did. 

There are several implications of this study. First, although FLS implementation in our open system improves care, the authors hypothesize that enhanced data-sharing between health systems would increase care further. This is a major advantage of closed systems that have been able to show a more robust effect of the FLS pathway. Second, when interventions are automated, as is the case for the FLS consult and is not the case for the flyer distribution, outcomes are improved. Lastly, follow up with an osteoporosis specialist is crucial for improving therapy initiation rates and for allowing patients access to a broader range of therapies which may be more effective in their individual case than oral bisphosphonates. 

The United States Health and Human Services has released the Healthy People 2030 initiative in 2025. Part of this initiative is to reduce hip fracture rates among older adults from 5.7 hospitalizations per 1,000 adults to 4.6 [23]. Additionally, National Committee for Quality Assurance/Healthcare Effectiveness Data and Information Set (NCQA/HEDIS) and Centers for Medicare & Medicaid Services (CMS) quality programs measure rates of bone mineral density testing or osteoporosis treatment initiation within 180 days of fracture as a measure of quality care [24,25]. Interventions like FLS programs to reduce the re-fracture rate should move the needle toward meeting the national standards listed above. Our study showcases a real-world FLS model in a system with over 10 possible insurance plans with hundreds of iterations (Preferred Provider Organization (PPO) vs. Health Maintenance Organization (HMO), Medicare Advantage Plans, Medicaid contractors, etc.) with a large catchment area as one of only two level one trauma centers in county of 2.214 million people. The setting of this study increases its generalizability. 

This study has several important limitations. First, this was not a randomized controlled trial, and the selection into outpatient follow-up, particularly specialist care, was non-random and influenced by prior system affiliation and access to care. Second, loss to follow-up was substantial, particularly in this open healthcare system, and treatment initiation may be underestimated due to incomplete capture of care occurring outside the interoperable EHR network, despite conservative non-responder imputation. Third, reliance on EHR documentation limits ascertainment of medication dispensing and adherence, as therapy initiation was defined by prescription or order entry rather than confirmed use. Fourth, the flyer intervention was designed as a quality-improvement pilot rather than a powered efficacy trial, and the modest sample size limits the ability to detect small differences in treatment initiation. The flyer was written at a 10th grade reading level, which is higher than what is typically recommended for written educational interventions, which may have affected its impact. Fifth, exclusion of patients lost to follow-up may introduce selection bias if these patients differed systematically in clinical severity. Sixth, the post-FLS observation period was truncated by the onset of the COVID-19 pandemic, limiting longer-term evaluation of program maturation and re-fracture outcomes. Finally, this was a single-center study in a large academic open system, which may limit generalizability to other healthcare settings.

## Conclusions

In summary, system-level implementation of an FLS in an open healthcare system increased the likelihood of osteoporosis treatment initiation when compared to pre-FLS care, although treatment rates remained low. Post-hospital rheumatology outpatient care predicted initiation of therapy. Current guidelines recommend anabolic therapy for post-fracture osteoporosis care. This was utilized by none of the treated patients who saw a PCP, and in a limited number of patients who saw a rheumatologist. Patient-level flyer handout implementation did not significantly improve outcomes, supporting the fact that system-level intervention is needed to improve outcomes in post-fracture care. Closing the osteoporosis secondary prevention treatment gap requires better interoperability between the EHRs of more health systems to allow for continuity of care.
